# “Sewer Gas and Its Dangers,” Letter from Prof. F. H. Hamilton

**Published:** 1882-08

**Authors:** A. R. Davidson


"SEWER GAS AND ITS DANGERS."—LETTER FROM PROF. F. H. HAMILTON.

NEW YORK, 43 W. 32d Street, July 26th, 1882.

EDITORS BUFFALO MEDICAL AND SURGICAL JOURNAL:

I desire to correct a statement made by Dr. Davidson in his paper on "Sewer Gas, and its Dangers," contained in the July number of your Journal.

Referring to my paper read before the New York Academy of Medicine, March 16, 1882, entitled the "Struggle for Life against Civilization and Æstheticism," Dr. Davidson says: "Dr. Hamilton declares that 'the foul gases readily pass through water, and there is no security against sewer gas but the proper use of chemicals, supplied to the traps daily;'" and then Dr. Davidson adds, "perhaps recognizing the impossibility of carrying out such a recommendation, he farther advises as the only real security, the placing of all the plumbing fixtures in an annex to the building. Dr. Hamilton's views were endorsed by Dr. Doremus."

The fact is that, so far as the use of chemicals was concerned, the remark attributed to me is substantially what was said by Prof. Doremus, and of which I expressed neither approval nor disapproval; nor did Prof. Doremus say one word by the way of approval or disapproval of what I said about placing the fixtures in an annex.

Probably Dr. Davidson was misled by some newspaper report, but if he will look at the report of my lecture as published in the *Medical Gazette*, where alone it was published entire, he will see how great has been his misunderstanding of the facts, and that there was no occasion for the intimation of either mental or moral weakness on my part, conveyed in the last clause of the above quoted paragraph; and that I did not in a written lecture, read before intelligent men, advise the public to do what I knew was "impossible," and then seek to escape from the dilemma in which I had deliberately placed myself, by advising the public to do some other thing which it was possible to do.

Yours truly, FRANK H. HAMILTON.

In an editorial in the *Sanitary Engineer* on "The Discussion of the Academy of Medicine on Plumbing," the following sentences appear:

"He (Dr. Hamilton) said, it was well known that water was no protection against these foul gases, for they readily passed through it. There was no remedy, he said, against sewer gas, except the proper use of chemicals supplied to the traps daily. He believed that, to secure the best protection, all plumbing fixtures should be placed in an annex to the dwelling."

Prof. Doremus is reported as saying, "He *also* believed that the only means of protection was by the use of chemicals in the trap."

When Prof. Hamilton's article appeared, I read it with much interest, but unfortunately failed to make a note of the journal in which it was, and it proved impossible to find when again wanted. The impression left upon my mind was, that Dr. Hamilton's views were fairly represented as above. On receipt of his letter I referred to the files of the *Medical Gazette* and find that the sentences objected to appear in Dr. Hamilton's article substantially as quoted, *but only* as the expressed opinion of Drs. Doremus and Parker, and without any expression "of approval or disapproval" on the part of Dr. Hamilton. My apologies are therefore due and are gladly made to Dr. Hamilton for the unintentional misrepresentation.

A. R. DAVIDSON.

